# STAT3 as a biologically relevant target in H3K27M-mutant diffuse midline glioma

**DOI:** 10.18632/oncotarget.28516

**Published:** 2023-10-04

**Authors:** Jacob B. Anderson, Samantha M. Bouchal, Liang Zhang, David J. Daniels

**Keywords:** H3K27M, DMG, DIPG, midline glioma, STAT3

Pediatric H3K27M-mutant diffuse midline gliomas (DMGs), including those formerly classified as diffuse intrinsic pontine gliomas (DIPG), are uniformly lethal CNS malignancies [1]. Children diagnosed with these tumors have an extremely poor prognosis, with a median survival of approximately 12 months [2]. The current standard of care for DMG includes possible biopsy for diagnostic confirmation and a 6-week course of palliative radiation [3, 4]. Despite enormous effort toward the development of novel therapeutics in DMG, chemotherapy remains ineffective in this disease. Indeed, over 100 clinical trials for chemotherapeutics in DMG have failed to show therapeutic benefit [5].

**Figure 1:**
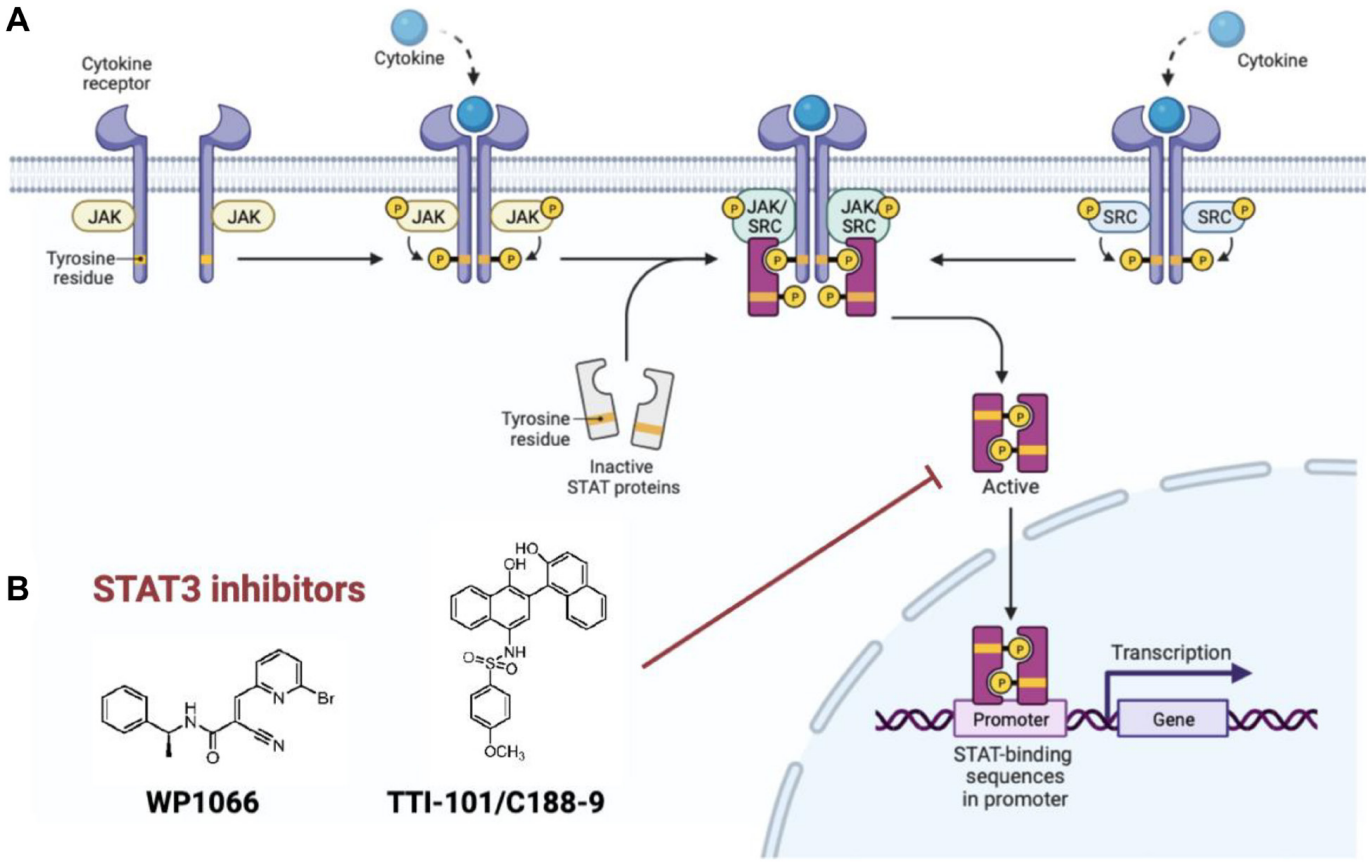
(**A**) Canonical STAT3 activation pathway. (**B**) WP1066 and TTI-101, examples of small molecule STAT3 inhibitors that are cell penetrant.

The family of transcription factors known as the Signal Transducer and Activator of Transcription (STAT) proteins are activated by extracellular signals such as growth factors and cytokines; these proteins are implicated in survival, proliferation, and differentiation at baseline [6]. STAT3, which is important in the cellular response to IL-6 and related cytokines, has long been known to contribute to tumor cell proliferation, growth, and evasion of apoptosis in cancer [7, 8]. Until recently, however, STAT3 was not a druggable target. Canonically, STAT3 is recruited from the cytosol to the cell membrane, where it interacts with different cytokine receptors through its Src homology 2 (SH2) domain. This results in phosphorylation of its Tyr705 residue (Y705), followed by dimerization and translocation into the nucleus for gene transcription (Figure 1A) [9]. Thus, in order to successfully and directly block STAT3 activity, an inhibitor must achieve blockade of STAT3 phosphorylation, dimerization, nuclear translocation, or STAT3-DNA binding activity. To date, STAT3 inhibitors have included oligonucleotides, peptidomimetics, and small molecule inhibitors [10]; however, some of these therapies present drug delivery challenges. For example, though peptidomimetics of phosphotyrosine are capable of binding tightly to the SH2 domain, thereby preventing STAT3 phosphorylation/dimerization in *ex vivo* experiments, they are often unable to effectively cross the cell membrane due to their large size and charge. Smaller, cell permeable STAT3 inhibitors often utilize covalent binding mechanisms to enhance target binding affinity and inhibition (Figure 1B) [11].

As detailed in our recently published manuscript, STAT3 is a biologically relevant therapeutic target in H3K27M-mutant diffuse midline glioma [12], our lab performed a screen of drugs currently in clinical use or clinical trials for efficacy against a library of H3K27M-mutant and H3-wildtype patient-derived cell lines. The results of this drug screen identified the STAT3 signaling pathway as a novel target in DMG. We demonstrated that phospho-tyrosine-705 STAT3 (pSTAT3) was selectively upregulated both in DMG patient specimens and patient-derived cell lines. We also showed that STAT3 inhibitors were markedly cytotoxic to H3K27M-mutant DMG *in vitro* and induce a robust restoration of H3K27 trimethylation (H3K27me3) expression which is normally absent in DMG tumors. Finally, the STAT3 pathway inhibitor WP1066, which utilizes a reversable-covalent mechanism to enhance target binding and inhibition [13], increased overall survival and decreased tumor growth in a patient-derived intracranial xenograft model of DMG.

Successful translation of STAT3 inhibitors to the clinic, both in DMG and in other cancers, will primarily require management of off-target effects and associated toxicity. Pathway inhibitors of STAT3 are currently in clinical trials for multiple CNS malignancies. A recent Phase I clinical trial of WP1066 in recurrent GBM found that this inhibitor is able to reduce STAT3 phosphorylation in peripheral blood with a 1 mg/kg dose [14]. In addition to the GBM Phase I trial, WP1066 also recently completed a Phase I clinical trial for children with progressive or recurrent malignant brain tumors (NCT04334863). Another actively recruiting trial is examining the efficacy of the STAT3 inhibitor silibinin in preventing recurrence of brain metastasis (NCT05689619). Finally, the STAT3 inhibitor TTI-101 recently underwent a large Phase I trial in multiple advanced malignancies (NCT03195699), and remains in active trials for metastatic breast and liver cancer (NCT05384119, NCT05440708). These compounds represent an exciting new avenue for treating these devastating diseases.
